# Linking the Extended Autonomic System with the Homeostat Theory: New Perspectives about Dysautonomias

**DOI:** 10.3390/jpm14010123

**Published:** 2024-01-22

**Authors:** David S. Goldstein

**Affiliations:** Autonomic Medicine Section, Clinical Neurosciences Program, Division of Intramural Research, National Institute of Neurological Disorders and Stroke, National Institutes of Health, Bethesda, MD 20892, USA; goldsteind@ninds.nih.gov; Tel.: +1-301-675-1110; Fax: +1-301-402-0180

**Keywords:** autonomic, dysautonomia, homeostat, homeostasis, allostasis

## Abstract

Dysautonomias are conditions in which altered functions of one or more components of the autonomic nervous system (ANS) adversely affect health. This essay is about how elucidating mechanisms of dysautonomias may rationalize personalized treatments. Emphasized here are two relatively new ideas—the “extended” autonomic system (EAS) and the “homeostat” theory as applied to the pathophysiology and potential treatments of dysautonomias. The recently promulgated concept of the EAS updates Langley’s ANS to include neuroendocrine, immune/inflammatory, and central components. The homeostat theory builds on Cannon’s theory of homeostasis by proposing the existence of comparators (e.g., a thermostat, glucostat, carbistat, barostat) that receive information about regulated variables (e.g., core temperature, blood glucose, blood gases, delivery of blood to the brain). Homeostats sense discrepancies between the information and response algorithms. The presentation links the EAS with the homeostat theory to understand pathophysiological mechanisms of dysautonomias. Feed-forward anticipatory processes shift input–output curves and maintain plateau levels of regulated variables within different bounds of values—“allostasis”. Sustained allostatic processes increase long-term wear-and-tear on effectors and organs—allostatic load. They decreaseing thresholds for destabilizing and potentially fatal positive feedback loops. The homeostat theory enables mathematical models that define stress, allostasis, and allostatic load. The present discussion applies the EAS and homeostat concepts to specific examples of pediatric, adolescent/adult, and geriatric dysautonomias—familial dysautonomia, chronic orthostatic intolerance, and Lewy body diseases. Computer modeling has the potential to take into account the complexity and dynamics of allostatic processes and may yield testable predictions about individualized treatments and outcomes.

## 1. Introduction

Dysautonomias are conditions in which altered functions of one or more components of the autonomic nervous system (ANS) adversely affect health. 

These disorders are frustrating, not only for patients but also for clinicians and researchers. There are several reasons for this. First, dysautonomias come in many forms—there is a whole “universe” of dysautonomias—that can involve essentially all body organs and systems. Because of this multiplicity and the multi-system and therefore multi-disciplinary nature of dysautonomias, they fall through the cracks of the traditional biomedical enterprise. Second, dysautonomias are complex, involving abnormalities in regulation of many effectors and organs by numerous brain anatomic and neurochemical networks. Third, dysautonomias seem often to be mind-body disorders that entail two-way miscommunications between the central autonomic network and body organs; this perspective flies in the face of the traditional Cartesian duality separating the psyche and soma. Fourth, different centers offer diverse autonomic function tests, with the repertoires seeming to heavily depend importantly on cost and throughput, insurance coverage, and regulatory constraints as opposed to tailoring the testing based on relevance to the assessment of individual patients. Fifth, and probably most significant, compared to the large and seemingly increasing patient demand and public health burden, clinical and basic training and scientific knowledge about dysautonomias are disproportionately sparse. This is the “grand challenge” of autonomic disorders [1].

The overall goal of this presentation is to inform the conversation about mechanisms of dysautonomias that may rationalize personalized treatments. Given the above difficulties in the field, one embarks on this sort of essay with some trepidation. Emphasized here are two relatively new ideas—the “extended” autonomic system (EAS) [2] and the “homeostat” theory [3] as applied to the pathophysiology and potential treatments of dysautonomias. I will be considering examples from “galaxies” in the dysautonomias universe, corresponding to pediatric, adult, and geriatric disorders. 

## 2. The “Extended” Autonomic System (EAS)

By mediating automatic, unconscious, involuntary behaviors, the ANS operates at the border of the body and mind. More than a century ago, the English physiologist John Newport Langley defined the ANS as consisting of three parts—the sympathetic nervous system, the parasympathetic nervous system (a phrase he coined), and the enteric nervous system [4]. These were thought to be purely efferent systems for transmitting neuronal signals via ganglia to body organs. 

**Figure 1:**
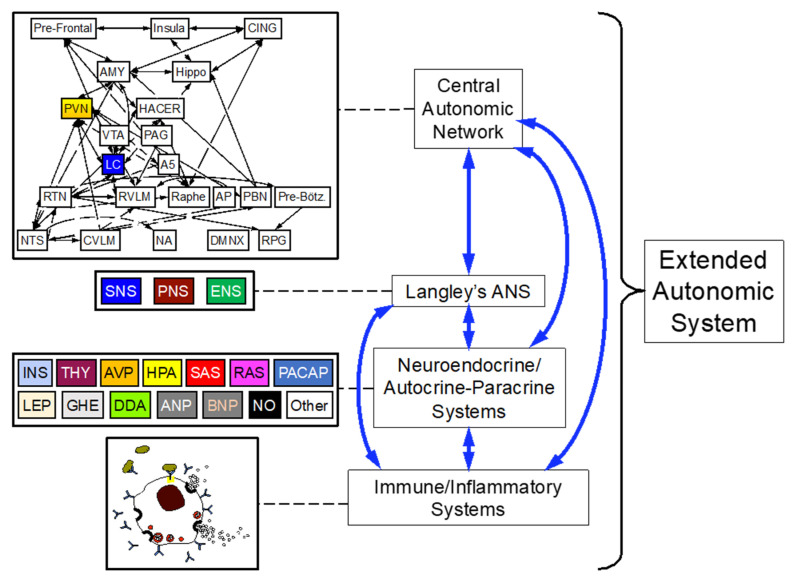
The extended autonomic system. The EAS consists of the central autonomic network (CAN)/stress system, Langley’s autonomic nervous system (ANS), neuroendocrine systems, and inflammatory/immune systems. The four components are bi-directionally inter-related, meaning 6 combinations of relationships. In the central autonomic network, the “stress system” in the Chrousos/Gold schema includes the paraventricular nucleus of the hypothalamus (PVN), which is the source of arginine vasopressin (AVP) and corticotrophin-releasing hormone that drive the hypothalamic-pituitary-adrenocortical axis (HPA), and the pontine locus ceruleus (LC), the main source of norepinephrine in the brain. Abbreviations: Abbreviations: A5 = A5 noradrenergic cell group; AMY = amygdala; ANP = atrial natriuretic peptide; ANS = autonomic nervous system; AP = area posterma; BNP = brain-derived neurotrophic factor; CING = cingulate cortex; CVLM = caudal ventrolateral medulla; DDA = DOPA-dopamine autocrine-paracrine system; DMNX = dorsal motor nucleus of the vagus nerve; ENS = enteric nervous system; GHE = ghrelin; HACER = hypothalamic area controlling emotional responses; Hippo = hippocampus; INS = insulin; LEP = leptin; NA = nucleua ambiguus; NO = nitric oxide; NTS = nuclear of the solitary tract; PACAP = pituitary adenyl cyclase-activating polypeptide; PAG = periaqueductal grey region; PNS = parasympathetic nervous system; Pre-Bötz = pre-Bötzinger complex; RAS = renin-angiotensin-aldosterone system; RPG = respiratory pattern generator; RTN = retrotrapezoid nucleus; RVLM = rostral ventrolateral medulla; SAS = sympathetic adrenergic system; SNS = sympathetic noradrenergic system; THY = thyroid; VTA = ventral tegmental area.

In the intervening century, three types of discoveries have rendered inadequate Langley’s theory of the ANS. First, in addition to neurotransmitter systems, a large number of endocrine and neuroendocrine systems mediate automatic, unconscious, involuntary activities within the body’s “inner world” [5]. One may reasonably contend that epinephrine was the first hormone and neuroendocrine effector to be identified [6]. Second, ANS components interact complexly and dynamically with immune and inflammatory systems [7]. Third, a brain network that is being described in increasing detail—the central autonomic network [8]—receives and integrates afferent signals from the periphery and modulates autonomic outflows. Based on these considerations, the recently disseminated concept of the “extended” autonomic system (EAS) expands on Langley’s ANS to include neuroendocrine, immune/inflammatory, and central facets (Figure 1).

## 3. The Homeostat Theory

Claude Bernard and Walter B. Cannon (who coined the term “homeostasis”) conceptualized that the overall “purpose” of body processes is to maintain the constancy of the internal environment. In contrast, in systems biology, homeostasis is more of an outcome than a goal [9].

**Figure 2:**
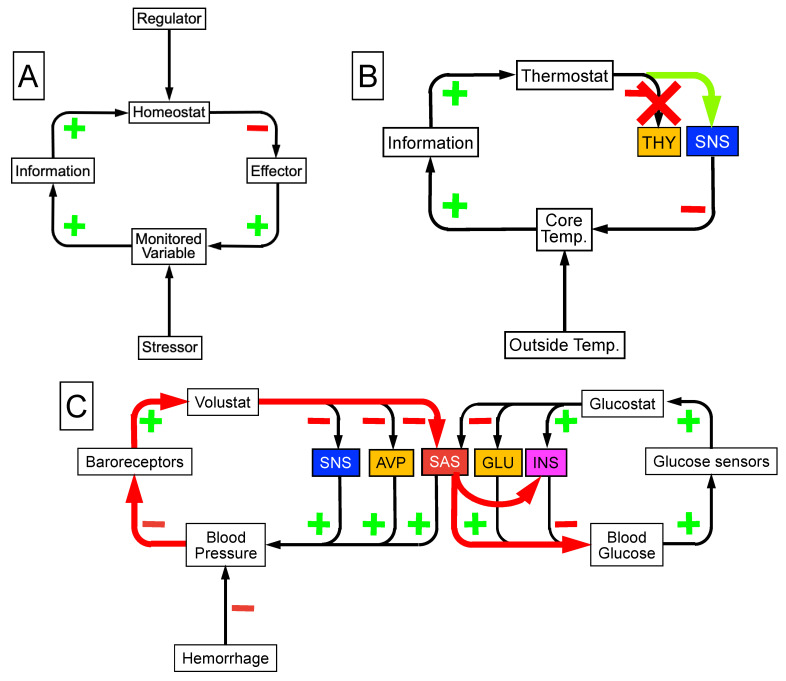
Principles of homeostat operation. Levels of the monitored variable are kept within bounds by negative feedback regulation (**A**). Negative feedback loops are characterized by an odd number of inhibitory processes (red (−) signs and arrows). Positive relationships are denoted by green + signs. A homeostatic comparator (homeostat) compares afferent information with a set point or other algorithm for responding. The discrepancy drives one or more effectors. (**B**) An example of compensatory activation when there are multiple effectors and one is disabled. Both the hypothalamic-pituitary-thyroid axis and the sympathetic noradrenergic system (SNS) are effectors for regulating core temperature (Core Temp.). Disruption of the hypothalamic-pituitary-thyroid axis compensatorily activates the SNS. (**C**) The sympathetic adrenergic system (SAS) is an effector that is shared by the barostat and glucostat. Effector sharing explains hyperglycemia in hemorrhagic shock. Abbreviations: AVP = arginine vasopressin; GLU = glucagon; INS = insulin.

The homeostat theory builds on Bernard’s and Cannon’s notions by proposing the existence of monitored, regulated variables (e.g., core temperature, blood glucose, blood gases, delivery of blood to the brain), which are controlled by comparator “homeostats” (e.g., thermostat, glucostat, carbistat, barostat) that sense discrepancies between afferent information and set points for responding (Figure 2).

Homeostats are metaphorical constructs [9]. No one knows what the “purposes” or “goals” of homeostatic systems are. One can postulate the existence of numerous homeostats—an “osmostat” for serum osmolality, an “oxistat” for blood oxygen tension, a “volustat” for effective circulating blood volume, and even a “nocistat” for the experience of pain and a “psychostat” for the sense of equanimity vs. distress. The thought process is that if body variables are kept within bounds, there must be systems at play that are designed to achieve these goals. Systems biologic approaches seem to avoid flirting with this sort of teleological assertion. 

## 4. Allostasis and Allostatic Load

Much of integrative physiological research has focused on negative feedback regulation—reflexes. In humans, however, long-term homeostasis is importantly maintained via anticipatory, feed-forward processes [10] that temporarily shift input–output curves and bring levels of regulated variables to different values—“allostasis” [11]. 

At first glance, the notion that allostatic processes operate in anticipation of need would seem paradoxical. How can a response occur before the stimulus that would generate that response? This has been a basis for criticizing teleological thinking. Actually, allostatic adjustments can be explained readily by effects of instinct, imprinting, conditioning (both classical (Pavlovian) and operant (instrumental)), and conscious simulations. Examples of instinct in the operations of the EAS would be a person’s heart rate increasing as part of “central command” in anticipation of exercise, instinctive avoidance behavior evoked by visual [12] or olfactory [13] predator cues, and innate immune responses to a viral infection. An example of classical conditioning would be augmented tachycardia in anticipation of standing up in patients with postural tachycardia syndrome (POTS) [14] because of learned associations of previously neutral cues with unconditioned aversive stimuli, such as nausea, chest pain, and faintness evoked by orthostasis. An example of operant conditioning would be learning to avoid situations involving prolonged standing, because they are aversive. An example of reacting to conscious simulations would be eating an energy bar before running a mile. Recent animal experiments have begun to identify the specific central pathways and neurochemicals in these responses. In general, they correspond to components of the central autonomic network, although the boundaries of that network seem to require extension to the motor cortex [15] and nigrostriatal dopaminergic system [16].

One of the characteristic features of a viral illness such as COVID-19 is a low-grade fever. According to the allostasis concept, the fever is the result of adjustments in input–output curves for the sympathetic noradrenergic system (SNS), which regulates delivery of blood to the skin surface, and the sympathetic cholinergic system (SCS), which regulates sweating. These adjustments keep core temperature within bounds (“stasis”) but at a different level (“allo”). The EAS idea accounts for these allostatic adjustments resulting in fever in COVID-19, in that the EAS incorporates the immune/inflammatory systems and input to the brain from biochemical signals arising from those systems [3].

Allostatic adjustments ordinarily are temporary. For instance, after a viral infection is over, the low-grade fever dissipates. An integrative physiological explanation for dysautonomias is that the allostatic adjustments persist [3]. Levels of regulated variables are kept at new values. This comes at the costs of greater energy utilization, increased variability, and accelerated wear-and-tear on effectors and body organs (allostatic load). 

The homeostat theory offers the ability to define difficult entities such as stress and allostatic load in ways that can be modeled mathematically [17,18]. For instance, stress can be defined as the condition in which an error signal drives effectors that decrease the error signal, and allostatic load can be defined as the integrated wear-and-tear on the effectors and consequently on body organs. Among other things, this model predicts that stress can accelerate the accumulation of allostatic load sufficiently to precipitate positive feedback loops and organ failure.

## 5. Principles of Homeostat Operation

### 5.1. Multiple Effectors

Having multiple effectors (Figure 2B) offers obvious survival advantages. These include extending the range of control of the monitored variable, compensatory activation of alternative effectors, and stressor-specific patterning. Cannon’s view about how blood glucose is maintained included two opposing effectors, the “sympathico-adrenal” system and the “vago-insular” system [19]. If the “common variation” of the level of glycemia fell below a given value (70 mg% was listed), “sympathico-adrenal” activation would raise the glucose level; if the level of glycemia exceeded a given value (130 mg%), “vago-insular” activation would decrease the glucose level. Because of the opposing effectors, the glucose level would be kept within bounds across a range of common variation. 

Compensatory activation of alternative effectors enables at least some degree of control of the level of the monitored variable when another effector is disabled. Examples of compensatory activation abound in physiology and pathophysiology, such as recruitment of accessory neck muscles in asthma attacks and augmentation of sympathetic noradrenergic responses to stress in adrenalectomized individuals [20]. Longer-term forms of compensatory activation exemplify plasticity, such as the development of collateral circulation in the setting of coronary artery blockage and adaptive changes in locomotion in movement disorders.

Having multiple effectors probably also permitted the evolution of patterned responses to different stressors. For instance, cold exposure selectively activates the SNS, while glucoprivation selectively activates the sympathetic adrenergic system (SAS) [21]. 

### 5.2. Effector Sharing

Effector sharing occurs when two or more homeostatic systems share the same effector. A classic example is the arginine vasopressin (AVP) system. AVP not only is a vasoconstrictor but also, acting as the anti-diuretic hormone, is the body’s main effector in regulation of water balance and hence of serum osmolality. Sharing of the AVP effector by the “barostat” and “osmostat” explains why patients in shock can be hyponatremic. Similarly, sharing of the SAS effector by the barostat and “glucostat” explains why the patients are hyperglycemic (Figure 2C).

## 6. Homeostats at Work

The key elements of the homeostat theory—monitored variables, regulators, and homeostats—are in essence metaphors. Experimental observations over the last two decades, however, have increasingly elucidated how homeostatic systems operate and have generally supported the concepts of multiple effectors, effector sharing, negative feedback regulation, and allostasis. The following discussion focuses on regulation of core temperature, glucose, blood gases, and delivery of blood to the brain during orthostasis. 

### 6.1. Thermoregulation

**Figure 3:**
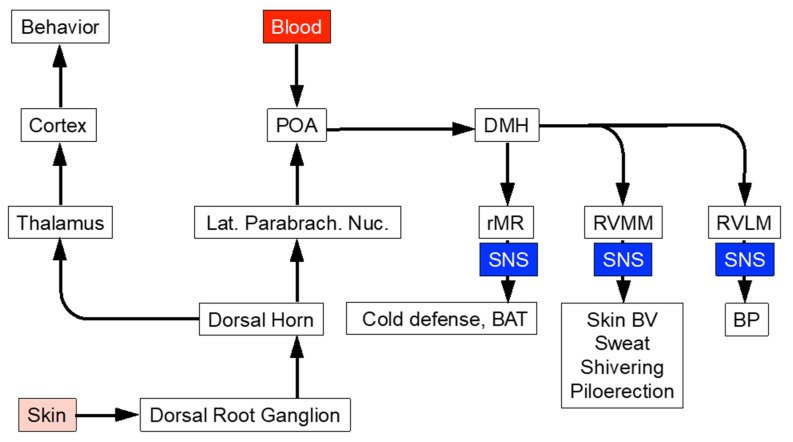
Central network controlling core temperature via the sympathetic noradrenergic system (SNS). Abbreviations: BAT = brown adipose tissue; BP = blood pressure; BV = blood vessels; DMH = dorsomedial hypothalamus; POA = pre-optic area; rMR = rostral medullary raphe; RVLM = rostral ventrolateral medulla; RVMM = rostral ventromedial medulla.

Humans have two primary sources of afferent information about temperature, the skin and the arterial blood (Figure 3). A neuronal pathway relays cutaneous sensory information via the dorsal horn, spinothalamic tract, and lateral brachial nucleus to the pre-optic area (POA), which also possesses neurons responsive to the temperature of the arterial blood. Subjective thermal comfort plays a critical role in body temperature regulation, since this represents the primary stimulus for behavioral thermoregulation. Core and skin temperature contribute about equally to thermal comfort, whereas metabolic heat production and plasma catecholamine responses are more responsive to core temperature [22].

In fruit flies, peripheral thermosensory information to higher brain centers converges onto three target regions: the mushroom body, the lateral horn, and the posterior lateral protocerebrum. Hot and cold antennal receptors project onto distinct but adjacent glomeruli in the proximal antennal protocerebrum, forming a thermotopic map in the brain. It has been proposed that “...dedicated populations of cells orchestrate behavioral responses to different temperature stimuli, and reveal a labeled-line logic for the coding of temperature information in the brain” [23].

### 6.2. Glucose

**Figure 4:**
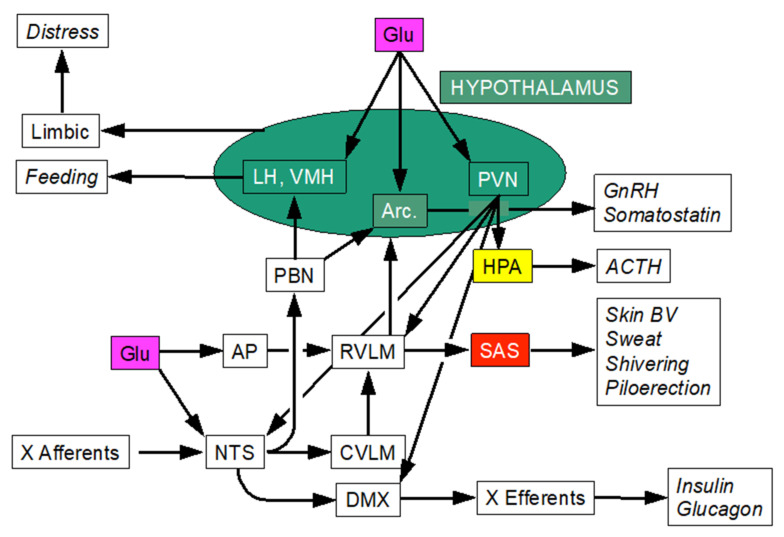
Central network controlling blood glucose. Abbrevations: AP = area postrema; Arc. = arcuate nucleus; BV = blood vessels; CVLM = caudal ventrolateral medulla; DMX = dorsal motor nucleus of the vagus nerve; GnRH = growth hormone-releasing hormone; HPA = hypothalamic-pituitary-adrenocortical axis; LH = lateral hypothalamus; NTS = nucleus of the solitary tract; PBN = parabrachial nucleus; PVN = paraventricular nucleus of the hypothalamus; RVLM = rostral ventrolateral medulla; SAS = sympathetic adrenergic system; VMH = ventromedial hypothalamic nucleus; X = vagus nerve.

The hypothalamic arcuate nucleus (ARC) is a key brain center for sensing adiposity signals (e.g., insulin, leptin, ghrelin, glucagon-related peptide 1) (Figure 4). ARC neurons not only regulate feeding but also contribute to glucose homeostasis and innate immune responses. 

Blood levels of glucose are regulated mainly by hormones, such as insulin from pancreatic islet β-cells, glucagon from pancreatic islet α-cells, epinephrine from adrenomedullary chromaffin cells, and, to a lesser extent, cortisol from adrenocortical zona fasciculata cells. These hormonal effects interact complexly. Glucagon may increase circulating glucose levels both directly via hepatic glucose release and indirectly via adrenomedullary epinephrine secretion [24]. Meanwhile, epinephrine stimulates pancreatic glucagon secretion [25], suggesting the potential for a self-reinforcing positive feedback loop. Epinephrine stimulates pancreatic insulin secretion via β-adrenoceptors but mainly inhibits insulin secretion via agonism at α-adrenoceptors. Epinephrine infusion blunts insulin responses to both hyperglycemia and glucagon [26]. 

Virtually every serious illness or cause of emotional distress is associated with hyperglycemia, even in individuals without a history of diabetes, and is associated with worse outcome [27,28,29,30,31]. One may reasonably propose that the adverse prognoses associated with hyperglycemia are not the result of hyperglycemia itself so much as of disease severity-related neuroendocrine changes producing hyperglycemia. 

### 6.3. Blood Gases

**Figure 5:**
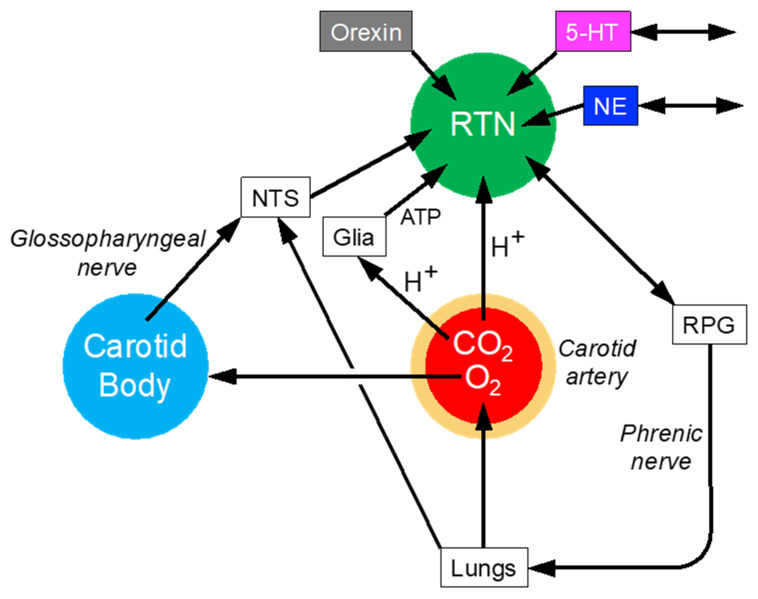
Central network controlling arterial blood gases.

In mammals, appropriate delivery of oxygen to and removal of carbon dioxide are crucial for survival. Multiple effectors for this regulation exist, and blocking one compensatorily activates others. The retrotrapezoid nucleus (RTN) neurons in the rostral ventrolateral medulla (RVLM) is part of a column of respiration-related neuronal clusters. The RTN is thought to regulate breathing automaticity and arterial pCO_2_ homeostasis (Figure 5). The carotid bodies stimulate the respiratory pattern generator both directly and indirectly by activating the RTN via a neuronal projection originating within the nucleus of the solitary tract (NTS). Consistent with the principle of multiple effects and compensatory activation, silencing RTN neurons increases carotid body activity [32]. 

### 6.4. Blood Flow to the Brain during Orthostasis

The requirements of correct core temperature and continuous availability of metabolic fuels have challenged organismic integrity throughout mammalian evolution. Multiple effectors have evolved to meet these challenges. In contrast, humans have been standing up since only relatively recently in evolutionary time. It is thought that in Africa, about 5–6 million years ago, there was a shift from jungle to savannah life. Bipedalism afforded obvious selective advantages in this new ecological niche, such as seeing further distances during migrating, carrying objects and infants, communication via hand gestures or arm waving, and more powerful striking and manipulating. According to cladographic data, our ancestor *Homo erectus* came on the scene only about 2–3 million years ago.

In order to tolerate standing, an individual must be able to tighten blood vessels below the level of the heart and increase the force and rate of cardiac contraction to maintain blood flow to the brain. One may speculate that because orthostasis is relatively new in evolutionary terms, only one system, the SNS, is available to maintain blood flow to the brain during orthostasis. Predictably, orthostatic intolerance and hypotension are cardinal manifestations of SNS failure.

**Figure 6:**
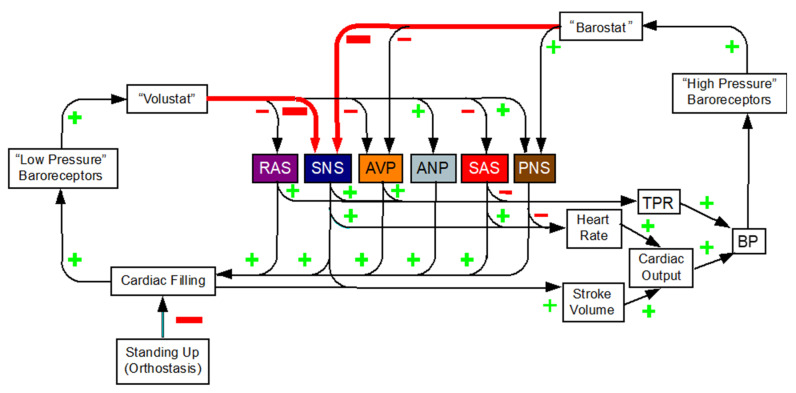
Low- and high-pressure baroreflexes. The sympathetic noradrenergic system (SNS) is the main effector for maintaining blood pressure (BP) during orthostasis. Diffuse SNS failure always manifests with orthostatic hypotension. Other effectors are the renin-angiotensin-aldosterone system (RAS), arginine vasopressin system (AVP), atrial natriuretic peptide (ANP), the sympathetic adrenergic system (SAS), and the parasympathetic nervous system (PNS).

There are two general types of afferent information to the brain during orthostasis (Figure 6). The first is high-pressure mechanoreceptors in the walls of arteries—especially in the carotid sinus, at the vascular gateway to the brain. The second is low-pressure mechanoreceptors in atria and pulmonary veins. Both types of mechanoreceptors are unloaded by the orthostatic decrease in venous return to the heart. 

The effectors mediating the homeostatic responses are similar, but there are some differences. Activation of the renin-angiotensin-aldosterone system seems to be more prominent with unloading of low- than of high-pressure mechanoreceptors [33]. Low-pressure mechanoreceptors also appear to play a prominent role in reflexive forearm vasoconstriction [34] and SAS activation [35].

Lower body negative pressure (LBNP) decreases venous return to the heart and simulates gravitational stress. Reflexive sympathetically-mediated vasoconstriction can explain maintenance of arterial blood pressure in this setting. Non-hypotensive LBNP decreases middle cerebral artery blood flow velocity without a change in arterial diameter [36]. These findings indicate that during orthostasis, brain blood flow decreases for the same level of blood pressure—operationally, an allostatic shift in the chair-shaped curve relating cerebral blood flow to blood pressure (autoregulation). One may hypothesize that individuals with relatively large orthostatic decreases in venous return to the heart would be more likely to have symptoms of orthostatic intolerance, such as lightheadedness or “brain fog”. Testing this hypothesis would require controlling for hyperventilation, which independently decreases middle cerebral artery blood flow velocity by decreasing arterial pCO_2_.

## 7. Application to Pediatric Dysautonomias: Familial Dysautonomia (FD)

Within the dysautonomias universe, the pediatric “galaxy” often entails substantial genetic load or embryological abnormalities in development of components of the ANS. 

A classic example is familial dysautonomia (FD), also referred to as Riley-Day syndrome and Type III hereditary sensory and autonomic neuropathy (HSAN III). FD is mainly a disease of people of Ashkenazic Jewish extraction, due to a founder effect; almost all the disease alleles share a common ancestral haplotype. The disease results from a splicing error in the Elongator acetyltransferase complex subunit 1 (*ELP1*) gene (also known as *IKBKAP*). The splicing error results in exon 20 being skipped in different tissues. 

The pattern of plasma levels of catechols in FD points to arrested development of sympathetic noradrenergic nerves, coupled with compensatorily increased activity of tyrosine hydroxylase and normal activity of the SAS [37]. FD patients have attenuated orthostatic increments in plasma norepinephrine levels [38], possibly reflecting a generalized abnormality of sensory afferents, including from mechanoreceptors [39]. 

FD patients are susceptible to crises of nausea and vomiting associated with tachycardia, sweating, hypertension, and behavioral changes. Cyclic vomiting in FD is associated with high circulating dopamine levels [40]. This hyperdopaminergic state seems to be pathophysiologically significant, because treatment with carbidopa, which inhibits catecholamine biosynthesis, is effective in mitigating the vomiting [41]. Vesicles containing newly synthesized norepinephrine are released preferentially during sympathetic stimulation [42], and acute increases in plasma dopamine are likely to reflect increased exocytotic release from sympathetic noradrenergic nerves. It is therefore reasonable to speculate that arrested development of sympathetic noradrenergic nerves in FD results in a form of functional dopamine-beta-hydroxylase deficiency and compensatorily increasing sympathetic traffic to extant terminals, so that during crises there is excessive dopamine release compared to the increases in plasma levels of norepinephrine and epinephrine. 

Multi-disciplinary management strategies have improved survival in FD. Experimental therapeutic efforts to treat the disease process itself have so far been unsuccessful. After development of an animal model of FD and high-throughput drug screening, the small molecule kinetin (6-furfurylaminopurine) seemed promising. The pharmaceutical development program ended in 2019 due to budgetary constraints and the rarity of the patient population. Other feasible therapeutic approaches are small nuclear RNA components [43] or antisense oligonucleotides [44] to treat the splicing defect. Also, gene replacement therapy has been proposed that would entail delivering Type 2 adeno-associated virus (AAV) to express a wild type copy of the *ELP1* gene [45] or Type 9 AAV for exon-specific inclusion of *ELP1* exon 20 in cells expressing the target pre-mRNA [46]. 

The most effective treatment for FD would be prevention of the disease. An effort is under way to avoid reproduction by heterozygous carriers [47]; theoretically, this might eventually eliminate the disease. 

## 8. Application to Adult Dysautonomias: Postural Tachycardia Syndrome (POTS)

Dysautonomias in adolescents or adults often involve complex, multi-system disorders of regulation of components of the ANS, where the effectors have developed normally. Chronic orthostatic intolerance in POTS and repeated episodes of neurocardiogenic syncope (NCS) involve many symptoms, such as fatigue, exercise and heat intolerance, presyncope, impaired concentration and memory, headache, coat hanger pain, early satiety, bloating or vomiting, tremulousness, and pallor. 

Both POTS and NCS are far more common in women than men, for reasons that remain poorly understood. Among vigorously healthy astronauts re-exposed to the earth’s gravity after prolonged space flight, orthostasis intolerance is far more prevalent in females. Application of a computer model of cardiovascular function has indicated that simple differences in physiognomy such as the longitudinal center of gravity can explain the greater prevalence of post-reentry orthostatic intolerance in women than men [48]. For the same orthostatic gravitational stress, women might have a greater shift in blood volume to pelvic veins and therefore a larger fall in venous return to the heart and cardiac stroke volume [49]. 

**Figure 7:**
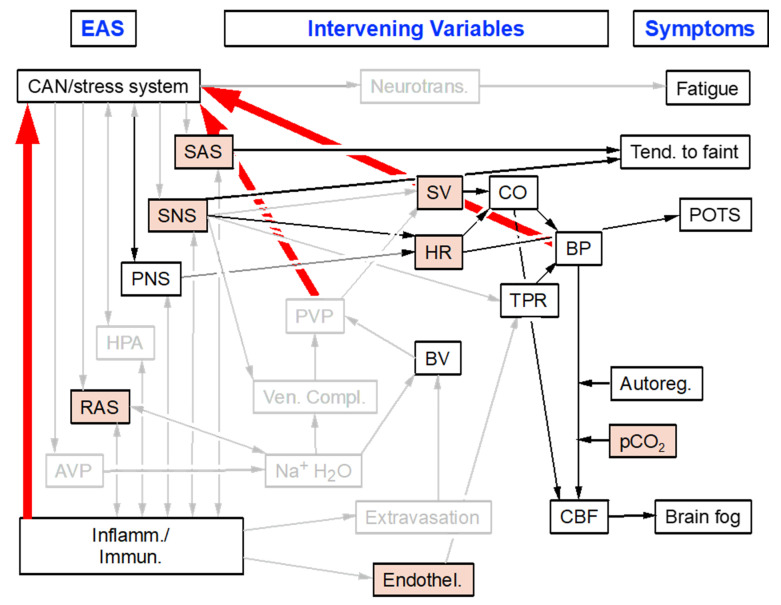
Concept diagram relating the EAS to intervening variables to symptoms of brain fog, a tendency to faint, and orthostatic intolerance in post-infectious POTS. Red arrows indicate afferent input to the brain from high-pressure and low-pressure mechanoreceptors. Grayed out boxes indicate variables for which objective data in POTS are incomplete or inconsistent. Pink filling indicates variables with abnormal values in POTS. Imbalance between sympathetic noradrenergic system (SNS) and sympathetic adrenergic system (SAS) outflows produces a tendency to faint. Other abbreviations: AVP = arginine vasopressin; Autoreg. = cerebrovascular autoregulation; BV = blood volume; BP = arterial blood pressure; CBF = cerebral blood flow; Endothel. = endothelial dysfunction; Neurotransm. = central neurotransmitters; POTS = postural tachycardia syndrome; PVP = pulmonary venous pressure; RAS = renin-angiotensin-aldosterone; SV = cardiac stroke volume; Tend. to faint = tendency to faint; TPR = total peripheral vascular resistance; Ven. Compl. = splanchnic venous compliance.

The schema in Figure 7 offers a concept for how neurocirculatory dyshomeostasis might result in persistent fatigue, a tendency to faint, excessive orthostatic tachycardia, and brain fog in POTS. The red arrows indicate afferent input to the central autonomic network from “high pressure” arterial baroreceptors that respond to alterations in systemic blood pressure, “low pressure” baroreceptors that respond to alterations in pulmonary venous pressure, and signals from the immune/inflammatory system. The numerous inter-relationships, most of which are bi-directional, seem dauntingly complex, yet they are derived from two relatively simple ideas, the EAS and the homeostat theory. 

In general, chronic orthostatic intolerance syndromes do not evolve to lethal neurodegenerative diseases, and in a substantial proportion of cases, the overall clinical status improves over time. Therapeutic interventions in which patients actively participate, such as graded exercise or counter-maneuvers [50], meditation, or yoga [51], might improve symptoms because of SNS activation in the setting of active coping [52].

## 9. Application to Geriatric Dysautonomias: Central Lewy Body Diseases (LBDs)

A major form of geriatric dysautonomias is a family of diseases involving Lewy bodies, intra-neuronal inclusion bodies having characteristic histopathological features. In Lewy body diseases (LBDs), Lewy bodies are found in brainstem dopaminergic and noradrenergic neurons or in sympathetic ganglia. Lewy bodies contain an abundance of the protein alpha-synuclein (αS). Conditions previously classified as forms of primary chronic autonomic failure—pure autonomic failure (PAF), multiple system atrophy (MSA), and Parkinson’s disease with orthostatic hypotension (PD + OH)—are referred to as autonomic synucleinopathies [53]. Dementia with Lewy bodies (DLB) involves a relatively high frequency of orthostatic hypotension and neuroimaging evidence of cardiac noradrenergic deficiency [54] and is now included in the family of autonomic synucleinopathies. All these disorders involve catecholamine deficiencies in the brain, the periphery, or both.

In the central LBDs PD and DLB, by the time parkinsonism or cognitive dysfunction manifests, clinically substantial catecholaminergic neurodegeneration has already occurred. Neurorescue strategies might forestall symptomatic disease if central LBDs could be identified in a preclinical phase. The prospective, observational, long-term PDRisk study assessed the predictive value of low vs. normal cardiac ^18^F-dopamine positron emission tomography (PET), an index of myocardial content of the sympathetic neurotransmitter norepinephrine [55] in at-risk individuals. At 7 years of follow-up, eight of nine participants with low initial ^18^F-dopamine-derived radioactivity and one of eleven with normal radioactivity were subsequently diagnosed with a central LBD (LBD+). Conversely, all of nine LBD+ participants had low radioactivity before or at the time of diagnosis of a central LBD, whereas among twenty-five participants without a central LBD, only one (4%) had persistently low radioactivity. Cardiac ^18^F-dopamine PET therefore highly efficiently distinguishes at-risk individuals who are subsequently diagnosed with a central LBD from those who are not [56]. These results have supported the view that the pathophysiological process leading to central LBDs can begin outside the brain, with early involvement of the autonomic nervous system—especially sympathetic noradrenergic innervation of the heart [55].

**Figure 8:**
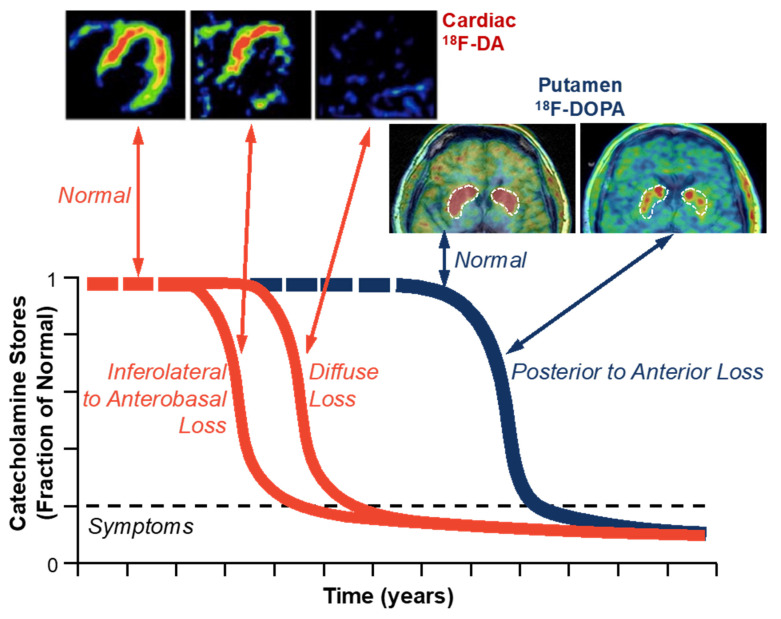
Tri-phasic loss of catecholamine stores in Lewy body diseases. Cardiac noradrenergic stores assessed by ^18^F-dopamine (^18^F-DA) positron emission tomography decline in a tri-phasic manner before tri-phasic decline in putamen ^18^F-DOPA-derived radioactivity. The loss of left ventricular myocardial ^18^F-DA-derived radioactivity proceeds from the inferolateral to the anterobasal wall, and the loss of putamen ^18^F-DOPA-derived radioactivity proceeds from the posterior to the anterior putamen.

Computational modeling has revealed multiple functional abnormalities in catecholaminergic neurons in LBDs [57]. These abnormalities can be explained by autotoxic interactions between oxidized metabolites of catecholamines and αS [58]. Extension of the modeling to address the trajectory of loss of catecholamine stores in LBDs over time has indicated a tri-phasic pattern [59] (Figure 8). For years, compensatory activation maintains homeostasis of striatal dopamine [60]. Once the compensatory processes are overwhelmed because of autotoxicity and allostatic load producing aging-related declines in efficiency, a second phase ensues in which there is a rapid decline in neurotransmitter stores (dyshomeostasis). When the complement of releasable catecholamine falls below a threshold level, the patient notes symptoms of the deficiency. In the symptomatic third phase, there is slow further loss.

The key to delaying the onset of symptomatic catecholaminergic neurodegeneration would be to begin treatment soon after the transition from homeostasis to dyshomeostasis. Mathematical modeling predicts that the same treatment that would exert only a small, transient benefit in symptomatic disease, but begun at the transition from homeostasis to dyshomeostasis, would substantially delay the onset of symptomatic disease [59].

It seems reasonable to propose that computational modeling, coupled with empirical data about EAS effectors and intervening variables, might yield testable hypotheses about exacerbating/ameliorating factors, responses to treatments, and outcomes in individual patients. Such a project, however, would require coordinating the efforts of integrative physiologists, systems biologists, and autonomic neuroscientists.

## 10. Conclusions

The EAS expands on the ANS by including neuroendocrine systems, immune/inflammatory systems, and the central autonomic network. The four components interact complexly and bi-directionally and determine clinical manifestations of dysautonomias. The homeostat theory enables objective, non-circular definitions of stress, allostasis, and allostatic load. Computer modeling has the potential to take into account the complexity and dynamics of allostatic processes [18,61] and may yield testable predictions about individualized treatments and outcomes.

## Data Availability

No new data were created or analyzed in this study. Data sharing is not applicable to this article.
